# Awareness of infective endocarditis prophylaxis in parents of children with congenital heart disease: A prospective survey

**DOI:** 10.4103/0974-2069.41057

**Published:** 2008

**Authors:** Parrimala Nath, V. Kiran, Sunita Maheshwari

**Affiliations:** Department of Pediatric Cardioloogy, Narayana Hrudayalaya Institute of Cardiac Sciences, Bangalore, India

**Keywords:** Infective endocarditis prophylaxis, congenital heart disease, parents, awareness

## Abstract

A prospective survey of parents of the children with congenital heart disesease was conducted to determine their awareness as regards the importance of oral hygiene and prophylaxis against infective endocarditis (IE). The results of this study demonstrated that only 8% of the parents were aware of the importance of good oro-dental hygiene and need for IE prophylaxis.

## INTRODUCTION

Heart disease is prevalent in children, and infective endocarditis (IE) is a potential complication in any child with heart disease. Although the fact that IE can occur in children with heart disease is well known among the medical fraternity, it is unknown how widespread this knowledge has been disseminated to the community, specifically to the parents of children with heart disease

A prospective verbal survey was therefore conducted in our hospital to assess how much and how many parents of children suffering from congenital heart diseases (CHDs) knew about the importance of and need for IE prophylaxis. The study period ranged from January 2005 to July 2006. Following three questions were asked to parents of 500 consecutive children known to be suffering from CHD.

1. How often does your child brush his/her teeth?
2. Do you know what to tell your child's dentist, if at all he/she has to see a dentist?
3. Are you aware of IE prophylaxis for your child?

The answers were then collected and analyzed.

Of the 500 children surveyed, 263 were boys and 237 were girls. The answers to the questions are detailed in Tables 1 and 2.

**Table 1:** Frequency of brushing the teeth (n=500)

Once	370	74%
Twice	107	21.4%
Irregular	20	4%
Never	3	0.6%

**Table 2:** Parents questioned regarding knowledge of IE prophylaxis (n=500)

Parents aware of IE prophylaxis	40
Parents unaware of IE prophylaxis	460

### Question 1:

Out of 500 children, 74% brushed their teeth once, 21.4% brushed twice, 4% were irregular in brushing and 0.6% never brushed their teeth at all

The visual assessment of oral cavity was done in all the children. They were classified as having good oral hygiene (normal oral cavity), fair oral hygiene (mild discoloration or plaque), or poor oral hygiene (visible caries, halitosis or gingivitis). On oral cavity examination, 141 children had good oral hygiene (28%); in 261 children, the oral hygiene was fair (52%) and in 98 children, it was poor (19%).

### Questions 2 and 3:

Out of the 500 parents, only 40 (8%) knew what they had to tell their child's dentist, if they had to visit him/her.

## DISCUSSION

Infective endocarditis is a potentially life-threatening condition despite several advances in its diagnosis and treatment. The estimated incidence of IE in the Western population has remained unchanged over the past two decades at 1.7-6.2 cases per 100,000 patient-years,[1–3] but such estimates are not available from India. Even assuming the lowest incidence, at least 17,000 episodes of IE must be occurring per year.[1,4–6]

A number of diagnostic and therapeutic procedures can cause transient bacteremia; hence, IE prophylaxis is very important. IE could be acute, wherein the course is rapid and fulminant with death occurring within 6 weeks if untreated. It is commonly caused by Staphylococcus aureus, Streptococcus pyogenes, and Streptococcus pneumoniae. The subacute IE has a slow, indolent course of several months duration, and is commonly caused by Streptococcus viridans.[7,8]

Since 1997, cardiac conditions were classified as high, moderate, and negligible risk groups and accordingly required IE prophylaxis. However, in October 2007, American Heart Association and European Cardiac Society came up with new recommendations for IE prophylaxis.[9] The pertinence of these guidelines in India is unclear as dental hygiene is suboptimal and presumably not at par with the Western standards. In this context, the earlier recommendations are still being followed in many centers, including ours.

There are very few studies[10,11] carried out regarding IE "awareness" and most of the studies showed that parental knowledge of IE prophylaxis is limited. These studies are from the 1980s and 1990s. Although the last two decades have seen significant technological advances, our study demonstrates that the basic management of children with heart disease still remains suboptimal.

The reasons for non-awareness of the need for good oral hygiene and IE prophylaxis could be poor socioeconomic status, illiteracy or ignorance among parents or a lack of education on the subject by medical personnel. Although we cannot change the socioeconomic and literacy status of families, one area where the medical community can effect a definite change is in education of families of children with heart disease. Education via pamphlets and via repeated mention of oral hygiene and IE prophylaxis at each clinic visit can help in building awareness among families.

## CONCLUSION

The results of this study demonstrated that only 8% of parents of children suffering from CHDs were aware of the need for IE prophylaxis and out of 500 children only 28% had good oral hygiene. Hence, a serious attempt has to be made by pediatricians and pediatric cardiologists to educate parents on importance of good oral hygiene the need for IE prophylaxis in children with heart disease.
